# The clinical significance of thyroid hormone-responsive in thyroid carcinoma and its potential regulatory pathway

**DOI:** 10.1097/MD.0000000000029972

**Published:** 2022-08-05

**Authors:** Zhen-xing Yu, Cheng Xiang, Sheng-gui Xu, Yang-ping Zhang

**Affiliations:** a Department of Thyroid Surgery, Mindong Hospital Affiliated to Fujian Medical University, Ningde, China; b Department of Thyroid Surgery, The Second Affiliated Hospital of Zhejiang University School of Medicine, Hangzhou, China; c Orthopedics Department, Mindong Hospital Affiliated to Fujian Medical University, Ningde, China.

**Keywords:** bioinformatics data, prognosis, thyroid carcinoma, thyroid hormone responsive expression

## Abstract

The study aimed to evaluate the clinical significance of thyroid hormone-responsive (THRSP) and explore its relevant pathways in thyroid carcinoma (THCA).

The gene expression data of THRSP were obtained and the prognostic significance of THRSP in THCA was analyzed through various bioinformatics databases. Then, the factors influencing THRSP mRNA expression were explored, and the function of THRSP in predicting the lymph node metastasis (LNM) stage was determined. We further performed the enrichment analysis and constructed a protein–protein interaction (PPI) network to examine potential regulatory pathways associated with THRSP.

THRSP gene expression was significantly increased in THCA compared with the normal tissues. High THRSP mRNA expression had a favorable overall survival (OS) in THCA patients (*P* < .05). Additionally, the mRNA expression of THRSP was related to stage, histological subtype, and methylation among THCA patients (all *P* < .05). Besides, THRSP served as a potent predictor in discriminating the LNM stage of thyroid cancer patients. According to Kyoto Encyclopedia of Genes and Genomes (KEGG) and gene set enrichment analysis (GSEA) on THRSP-associated genes, THRSP was positively related to metabolic pathways.

The upregulation of THRSP predicted a good OS in THCA patients. Furthermore, THRSP might inhibit THCA progression through positive regulation of metabolism-associated pathways.

## 1. Introduction

Thyroid carcinoma (THCA) is one of the most common cancers worldwide, and its morbidity is increasing rapidly.^[1,2]^ In the United States, THCA was the fifth most frequent malignancy in females. According to statistics, there were more than 53,000 new cases of THCA in 2020.^[3]^ In China, the incidence of THCA has also shown a rapid upward trend in recent years, which has increased by an average of 14.73% per year in men and 18.98% per year in women.^[4]^ THCA can be classified into papillary (80%–85% of all cases), follicular (10%–15%), medullary (<2%), and anaplastic (<2%) carcinomas.^[5]^ Although achievement has been made in diagnosing THCA at an early stage, the clinicians are faced with the challenge of the recognition of benign or malignant nodules, and the 5-year survival rate of THCA remains low.^[6]^ Lymph node metastasis (LNM) was an important indicator of THCA recurrence and metastasis. Accurate prediction of LNM is essential in selecting appropriate treatment methods, improving curative effects, and prolonging the survival time of patients.^[7]^ Although ultrasound is a convenient and effective method for thyroid disease screening, the efficacy of LNM detection by ultrasound is not ideal and occult LNM might be missed.^[8–10]^ Therefore, it is of great importance to identify new biomarkers for assisting the LNM prediction and improving THCA treatment.

The thyroid hormone-responsive (THRSP), also known as Spot 14, was a responsive regulator to thyroid hormones and was originally identified in the liver of fasted rats.^[11,12]^ It is a nuclear protein that is induced physiologically in lipogenic tissues including adipose, liver, and mammary gland, under circumstances that require brisk long-chain fatty acid synthesis including lactation in mammary epithelial cells.^[13]^ THRSP is a vital transcription factor and inhibition of its expression prevented activation of genes coding lipogenic enzymes, such as fatty acid synthase, ATP citrate-lyase, and acetyl CoA-carboxylase in hepatocytes.^[14,15]^ The THRSP gene regulation is mediated by a series of promoter elements that transduce signals triggered by dietary substrates, fuel-related hormones, and a negative regulatory element for polyunsaturated fatty acids.^[13]^ It has been demonstrated that THRSP promoted breast cancer cell growth, proliferation, and fatty acid synthesis. And the increased expression of THRSP predicted tumor recurrence and poor prognosis for patients with breast cancer.^[16,17]^ Besides, the elevated THRSP expression exerts a critical role in the pathogenesis of liver-related diseases by regulating lipid metabolism.^[18,19]^ The study of Hu et al proved that the reduced expression of THRSP in hepatocellular carcinoma had a positive relationship with a more aggressive phenotype, larger tumor size, and recurrence.^[20]^ In recent years, some genes have been proved to be crucial in the pathogenesis of THCA. For example, dysregulation of LDL receptor-related protein and aspartic acid [D]-rich C-terminal domain 1 was closely related to the THCA cell proliferation and migration.^[21,22]^ Sun et al showed that THRSP is one of 327 upregulated genes in the papillary THCA, which was validated by qPCR^[23]^; however, there has been no systemic research on the role of THRSP in THCA.

In this study, multiple databases were adopted to obtain the THRSP expression data. Then, we explored the prognostic value of THRSP and further identified the role of THRSP in predicting the LNM stage in THCA. Subsequently, the biological functions of THRSP and its potential regulatory pathways in THCA were investigated by enrichment analysis and protein–protein interaction (PPI) network construction.

## 2. Materials and Methods

### 2.1. Gene expression analysis

First, the transcriptional level of THRSP in pan-cancer was evaluated through Tumor Immune Estimation Resource database (https://cistrome.shinyapps.io/timer/), with statistical significance of differential expression using the Wilcoxon test. The THRSP mRNA expression in various cancer cell lines was explored in the Cancer Cell Line Encyclopedia database (https://portals.broadinstitute.org/ccle). Next, Gene Expression Profiling Interactive Analysis (GEPIA) (http://gepia.cancer-pku.cn/) and Oncomine (https://www.oncomine.org/resource/main.html) databases were used to assess the THRSP mRNA expression in THCA and normal tissues. The threshold in Oncomine database were THRSP gene, *P* value <.05, fold-change >2 and gene rank = top 10%.

### 2.2. Analysis of the clinical significance of THRSP

The effect of THRSP mRNA expression on the prognosis of THCA patients was investigated in terms of overall survival (OS) and disease-free survival (DFS) in the GEPIA database by setting parameters of “median” as the group cutoff.

### 2.3. Analysis of the factors affecting THRSP mRNA expression

To evaluate the impact of the clinicopathological parameters on THRSP mRNA expression, the data about patient clinical characteristics and THRSP expression were generated from the cBioPortal database (https://www.cbioportal.org/). We searched “THCA” and selected the “Thyroid Carcinoma (TCGA, Firehose Legacy)” dataset (n = 516). The samples with mapped clinical information and gene expression matrix were enrolled. Patients were divided into 2 expression groups according to the median expression of THRSP. The chi-squared test was performed to qualitatively analyze the relationship between THRSP mRNA expression and clinicopathological factors in THCA. The *t*-test was used to assess differential THRSP expression between N0 and N1 stages, and 1-way ANOVA was employed to test for differences among the 3 histological subtype groups. *P* < .05 was considered statistically significant.

Following this, the impact of genetic alteration and DNA methylation on THRSP mRNA expression was examined. cBioPortal was employed to determine the THRSP genetic alteration and its DNA methylation according to the “Thyroid Carcinoma” (TCGA, Firehose Legacy) dataset (n = 516). The THRSP genetic alteration was exhibited in the “OncoPrint” module and the association of THRSP mRNA expression with its DNA methylation status was presented in the “Plots” tab. Moreover, we compared the promoter methylation level of THRSP between normal and THCA groups using the UALCAN database (http://ualcan.path.uab.edu/). *P* value <.05 was considered to have a statistically significant difference.

### 2.4. Analysis of the value of THRSP in predicting LNM stage

Using TCGA-THCA data, logistic regression analysis was performed to explore the relationship between THRSP expression and the LNM stage. Receiver operating characteristic (ROC) analysis is a useful tool to evaluate the performance of diagnostic tests and to assess the accuracy of a statistical model such as logistic regression that classifies subjects into one of 2 categories.^[24]^ We performed ROC analysis to evaluate the value of THRSP expression in predicting the LNM stage. A *P* value <.05 was considered statistically significant.

### 2.5. Enrichment analyses of THRSP and its co-expressed genes

To elucidate the biological functions and the potential signaling pathways of THRSP involved in THCA, we performed Gene Ontology (GO) annotation and Kyoto Encyclopedia of Genes and Genomes (KEGG) pathways analyses. We first identified THRSP co-expressed genes in THCA through the LinkedOmics database (http://linkedomics.org/admin.php) which includes multi-omics data from all 32 TCGA cancer types and 10 Clinical Proteomics Tumor Analysis Consortium cancer cohorts. The following criterion was adopted for screening: Cancer cohort: TCGA_THCA; Dataset: TCGA_THCA (RNA seq); Dataset attribute: THRSP; Target dataset: TCGA_THCA (RNA seq); Statistical method: Spearman Correlation test. In accordance with the absolute value of *Z* score, the top 200 co-expressed genes were loaded into the DAVID database (https://david.ncifcrf.gov/tools.jsp) for the functional enrichment analysis. All the selected genes had *P* value <.001 and a false discovery rate *q* value <.001.

To reveal the underlying pathogenesis of THRSP in THCA, gene set enrichment analysis (GSEA) was performed in the WEB-based Gene Set Analysis Toolkit database (http://www.webgestalt.org/#) to explore THRSP-related positive and negative KEGG pathways. We inputted the selected 200 genes and THRSP into the database and set advanced parameters of the minimum number of genes for a category: 5; the maximum number of genes for a category: 500; the number of permutations: 1000. Gene sets with a false discovery rate *q* value <0.25 and *P* value <.05 were regarded as significant pathways.

### 2.6. PPI network construction

To determine the proteins associated with THRSP function and confirm potential pathways in which THRSP may be involved, the PPI network was constructed and significant KEGG pathways were acquired in the Search Tool for the Retrieval of Interacting Genes online database (https://string-db.org/). The database is widely used to identify the interactions between known proteins and predicted proteins, and to construct a PPI network. Those from curated databases and experimentally determined were known interactions. The predicted interactions included gene neighborhood, gene fusions, gene co-occurrence, and others such as textmining, co-expression, and protein homology.

### 2.7. Ethical statement

All data in this study were obtained from open public databases; we did not obtain these data from patients directly or intervene in these patients. Therefore, ethical approval was not required for this study.

## 3. Results

### 3.1. THRSP gene expression analysis

**Figure 1.**
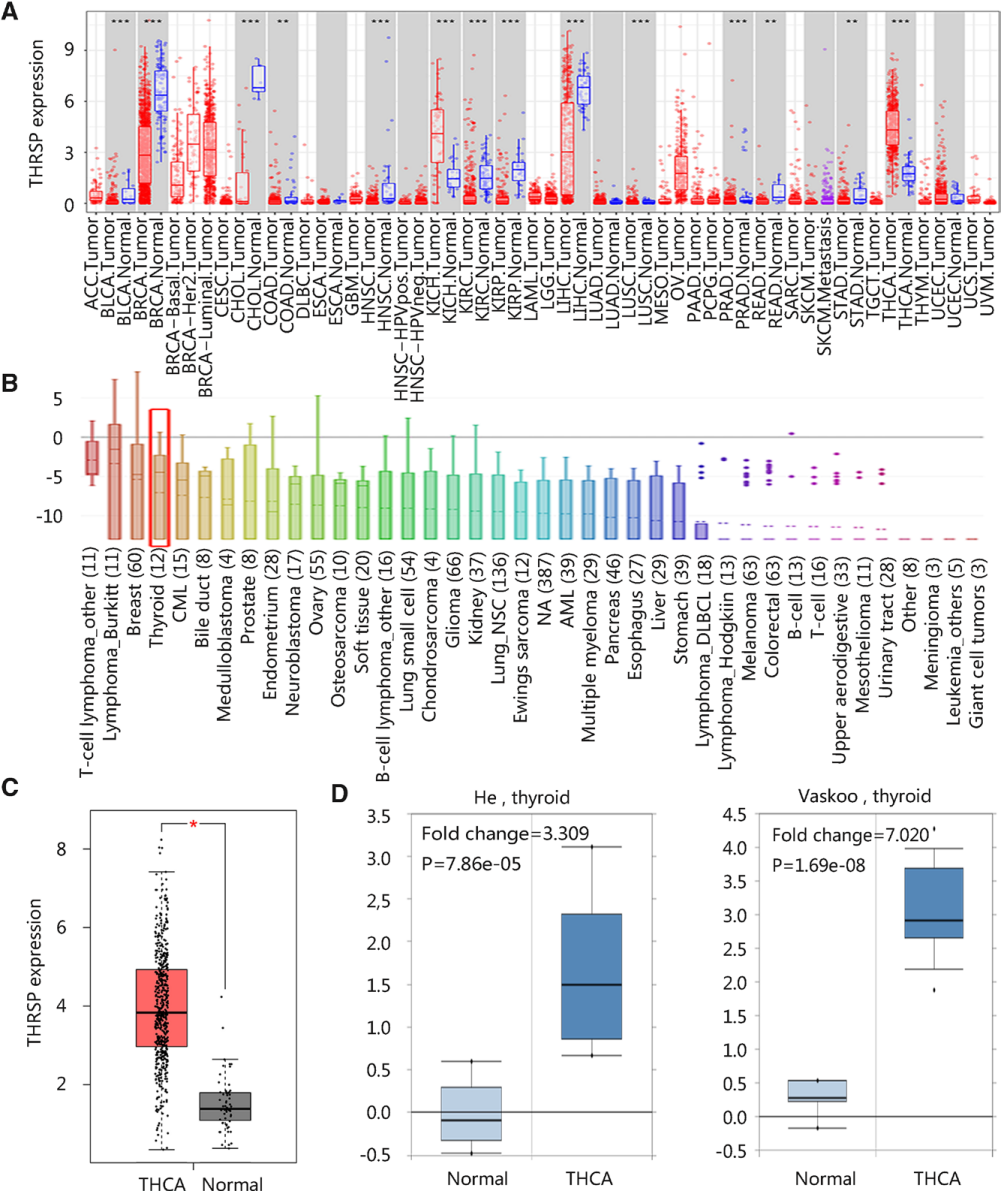
THRSP mRNA expression profile. (A) THRSP gene expression in various tumors and adjacent normal tissues. The red box indicates THCA and thyroid gland. (B) THRSP gene expression in different cancer cell lines. The red box indicates THCA cell lines. Differential expression analysis between THCA and normal tissues in (C) GEPIA, and (D) Oncomine. *P < .05; **P < .01; ***P < .001 compared with normal groups. THCA = thyroid carcinoma, THRSP = thyroid hormone-responsive.

Pan-cancer analysis showed that abnormal gene expression of THRSP was observed in 14 cancer types including THCA (all *P* < .01) (Fig. 1A). Besides, THRSP gene expression was relatively high in THCA cell lines (Fig. 1B). Group comparison revealed that the THRSP gene was highly expressed in THCA (*P* < .05; Fig. 1C). To further confirm the upregulation, the Oncomine database was employed to compare the THRSP gene expression in normal and THCA groups (*P* < .001; Fig. 1D).

### 3.2. The clinical significance of THRSP in THCA

**Figure 2.**
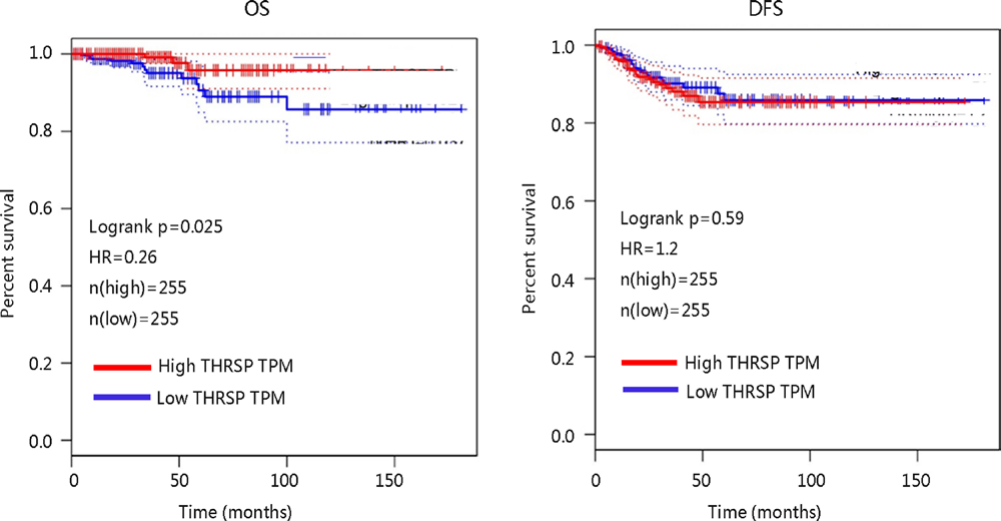
The effect of THRSP on OS and DFS in thyroid carcinoma via GEPIA. DFS = disease-free survival, GEPIA = Gene Expression Profiling Interactive Analysis, HR = hazard ratio, OS = overall survival, THRSP = thyroid hormone-responsive, TPM = transcription per million.

The GEPIA database was used to explore the effect of THRSP gene expression on the prognosis of THCA. Figure 2 showed that THCA patients with high THRSP transcription levels had longer OS time with statistical significance (*P* < .05). Interestingly, no significant difference was observed between the 2 THRSP expression groups with regard to DFS. These results suggested that THRSP might be a potential biomarker for predicting the OS of THCA patients.

### 3.3. Analysis of the factors affecting THRSP mRNA expression

**Figure 3.**
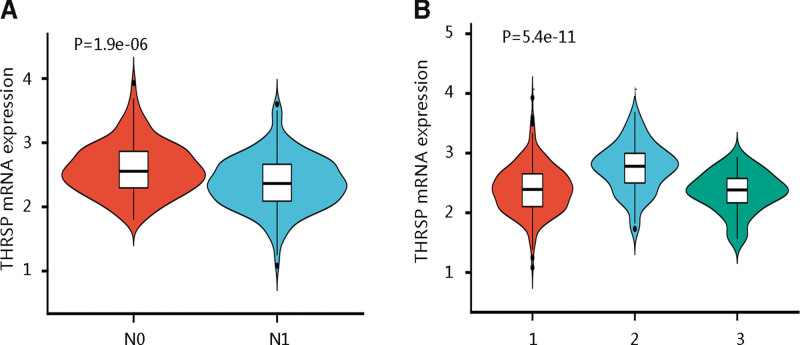
The significant association of THRSP expression with (A) lymph node metastasis stage and (B) histological subtype. 1 = papillary thyroid carcinoma (PTC)-classical, 2 = PTC-follicular, 3 = PTC-tall cell, N0 = no lymph node metastasis, N1 = lymph node metastasis, THRSP = thyroid hormone-responsive.

The above findings have revealed the prognostic significance of THRSP in THCA, we then assessed the clinical characteristics influencing the mRNA expression of THRSP. Age, race, and gender had no remarkable relationship with THRSP mRNA expression. However, the LNM stage and histological subtype were significantly related to the transcription level of THRSP in THCA (*P* < .001; Table 1). Of note, patients at the N0 stage had a higher THRSP mRNA expression than those at the N1 stage (*P* < .001; Fig. 3A). Among the histological subtypes, patients with the papillary THCA-follicular subtype had the highest THRSP gene expression (*P* < .001; Fig. 3B).

**Table 1:** Correlation between THRSP and clinicopathological factors in thyroid carcinoma.

Variables	THRSP expression			
	Low	High	χ*^2^*	*P* value
Age (yr)			0.551	.458
<55	134	127		
≥55	64	71		
LNM stage			22.382	<.001
N0	70	117		
N1	128	81		
Race			1.513	.469
African-American	9	6		
Asian	19	24		
Caucasian	139	124		
Gender			0.195	.659
Female	142	138		
Male	56	60		
Histological type			27.877	<.001
PTC-classical	158	128		
PTC-follicular	15	55		
PTC-tall cell	21	13		

**Figure 4.**
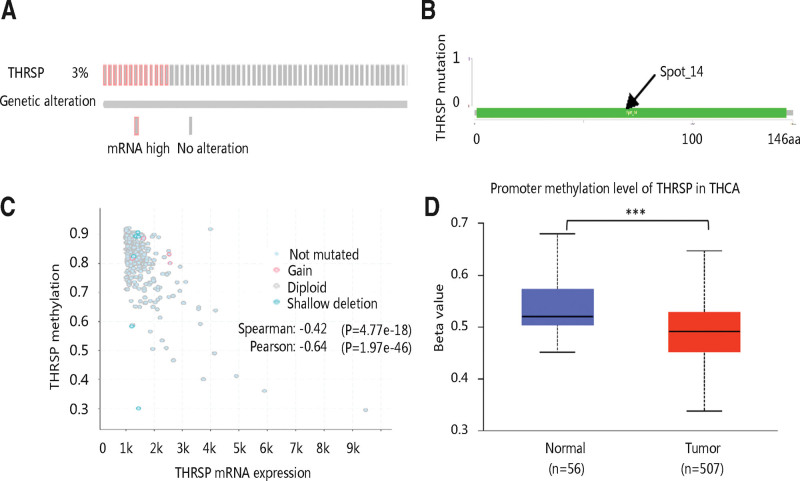
THRSP genetic alteration and DNA methylation in THCA. (A) THRSP genetic alteration. (B) THRSP mutation. (C) The relationship between THRSP methylation and THRSP mRNA expression. (D) The promoter methylation level of THRSP in normal thyroid gland and THCA tissues. The beta value indicates level of DNA methylation ranging from 0 (unmethylated) to 1 (fully methylated); ***P < .001. THCA = thyroid carcinoma, THRSP = thyroid hormone-responsive.

We further explored the mechanisms underlying aberrant mRNA expression of THRSP in THCA patients. Among 516 patients, 397 cases had THRSP genetic alteration and mutations measured. The results showed that mRNA high was the main form of genetic alterations which occurred in 3% of the patients, and no mutation was observed (Fig. 4A, B). However, THRSP mRNA expression was shown to have a strong negative relationship with its methylation (Fig. 4C). Then, we checked the promoter methylation level of THRSP in THCA and the normal thyroid gland. THCA group had a significantly lower THRSP methylation level compared with the normal group (*P* < .001; Fig. 4D). Therefore, the authors speculated that THRSP low methylation might lead to high THRSP mRNA expression, which needed to be verified in vitro and in vivo experiments in the future.

### 3.4. The value of THRSP in predicting the LNM stage

**Figure 5.**
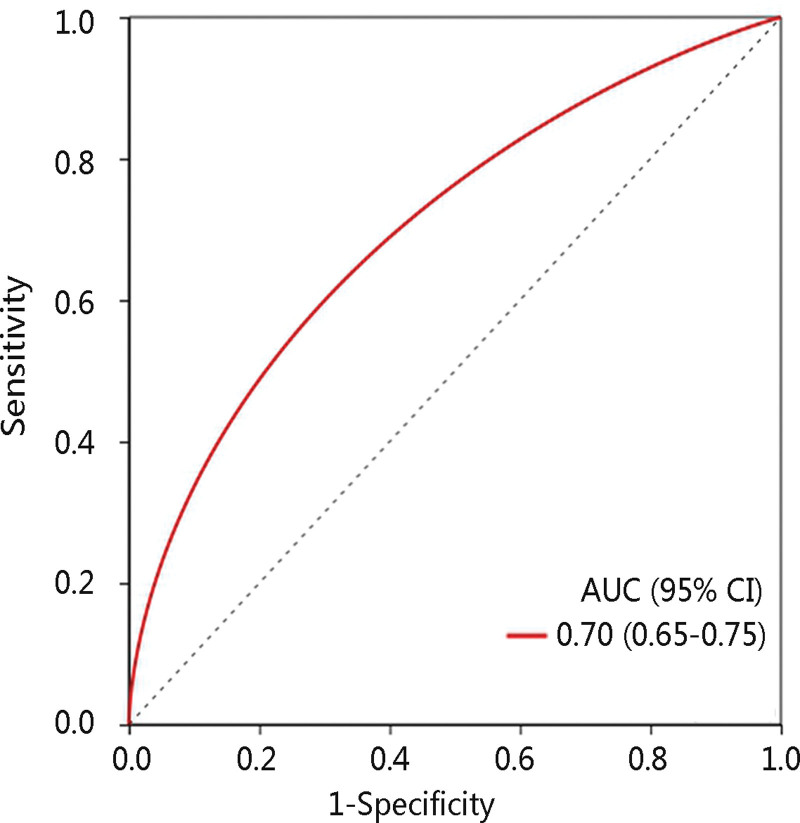
The receiver operating characteristic curve of THRSP in predicting the lymph node metastasis stage. 95% CI = 95% confidence interval, AUC = area under the curve, THRSP = thyroid hormone-responsive.

Since the LNM stage is an essential predictor of thyroid cancer prognosis and we have demonstrated that it was significantly related to THRSP mRNA expression, we next investigated its role in predicting the LNM stage. The logistic regression analysis result revealed that THRSP overexpression was related to low LNM risk with an odds ratio of 0.998 (*P* < .001). Moreover, the area under the curve (AUC) of the ROC curve of THRSP was 0.70 (Fig. 5), indicating the good performance of THRSP in distinguishing the LNM stage.

### 3.5. Enrichment analysis on THRSP and its co-expressed genes

**Figure 6.**
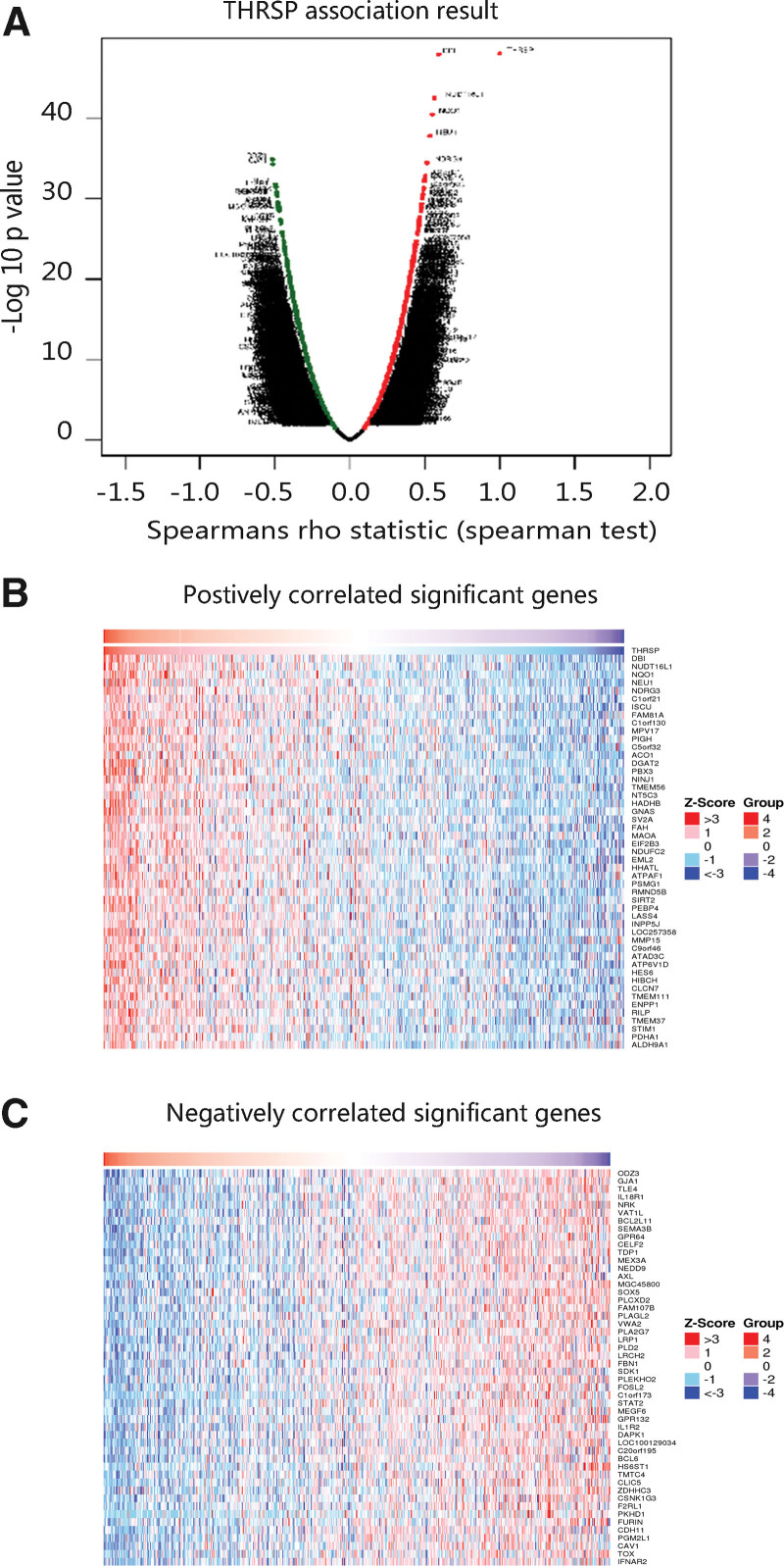
Co-expressed genes of THRSP in thyroid carcinoma from LinkedOmics. (A) Volcano plot of genes co-expressed with THRSP. (B) Top 50 positively correlated significant genes with THRSP. (C) Top 50 negatively correlated significant genes with THRSP. THRSP = thyroid hormone-responsive.

To disclose the biological function of THRSP and pathways in which THRSP might be involved, we first identified the genes co-expressed with THRSP in THCA through the LinkedOmics database according to the selection parameters (Fig. 6A–C). As shown in Table 2. The top biological processes were cellular calcium ion homeostasis, tricarboxylic acid cycle, cellular response to transforming growth factor-beta stimulus, positive regulation of the apoptotic process, and negative regulation of cell growth. The cellular components that THRSP mainly enriched in were extracellular exosome, an integral component of membrane, and mitochondrion. The major molecular functions were interleukin-1 receptor activity, receptor activity, and guanyl-nucleotide exchange factor activity. Besides, THRSP and its co-expressed genes had involvement in the biosynthesis of antibiotics, metabolic pathways, and carbon metabolism. The results revealed that THRSP may be related to tumor cell growth, and proliferation.

**Table 2:** GO and KEGG analyses of THRSP and its co-expressed genes in thyroid carcinoma.

Category	ID	Term	Count	*P* value
GO BP	GO:0006874	Cellular calcium ion homeostasis	6	1.90E-03
	GO:0006099	Tricarboxylic acid cycle	4	2.54E-03
	GO:0071560	Cellular response to transforming growth factor-beta stimulus	4	1.11E-02
	GO:0043065	Positive regulation of apoptotic process	8	2.43E-02
	GO:0006006	Glucose metabolic process	4	2.21E-02
	GO:0030308	Negative regulation of cell growth	5	2.80E-02
	GO:0045909	Positive regulation of vasodilation	3	3.06E-02
GO CC	GO:0070062	Exosome	49	3.96E-06
	GO:0016021	Integral component of membrane	62	7.22E-03
	GO:0005739	Mitochondrion	22	7.87E-03
	GO:0005765	Lysosomal membrane	8	1.18E-02
	GO:0005887	Integral component of plasma membrane	21	2.78E-02
	GO:0005829	Cytosol	40	3.89E-02
GO MF	GO:0004908	Interleukin-1 receptor activity	3	1.89E-03
	GO:0004872	Receptor activity	7	1.89E-02
	GO:0005085	Guanyl-nucleotide exchange factor activity	5	2.75E-02
	GO:0005089	Rho guanyl-nucleotide exchange factor activity	4	3.85E-02
	GO:0005509	Calcium ion binding	13	4.54E-02
	GO:0004871	Signal transducer activity	6	4.80E-02
KEGG	hsa01130	Biosynthesis of antibiotics	12	1.81E-05
	hsa01100	Metabolic pathways	30	1.83E-05
	hsa01200	Carbon metabolism	8	2.30E-04
	hsa00280	Valine, leucine and isoleucine degradation	5	1.71E-03
	hsa00020	Citrate cycle	4	4.25E-03
	hsa00630	Glyoxylate and dicarboxylate metabolism	3	3.54E-02
	hsa00640	Propanoate metabolism	3	3.78E-02

**Figure 7.**
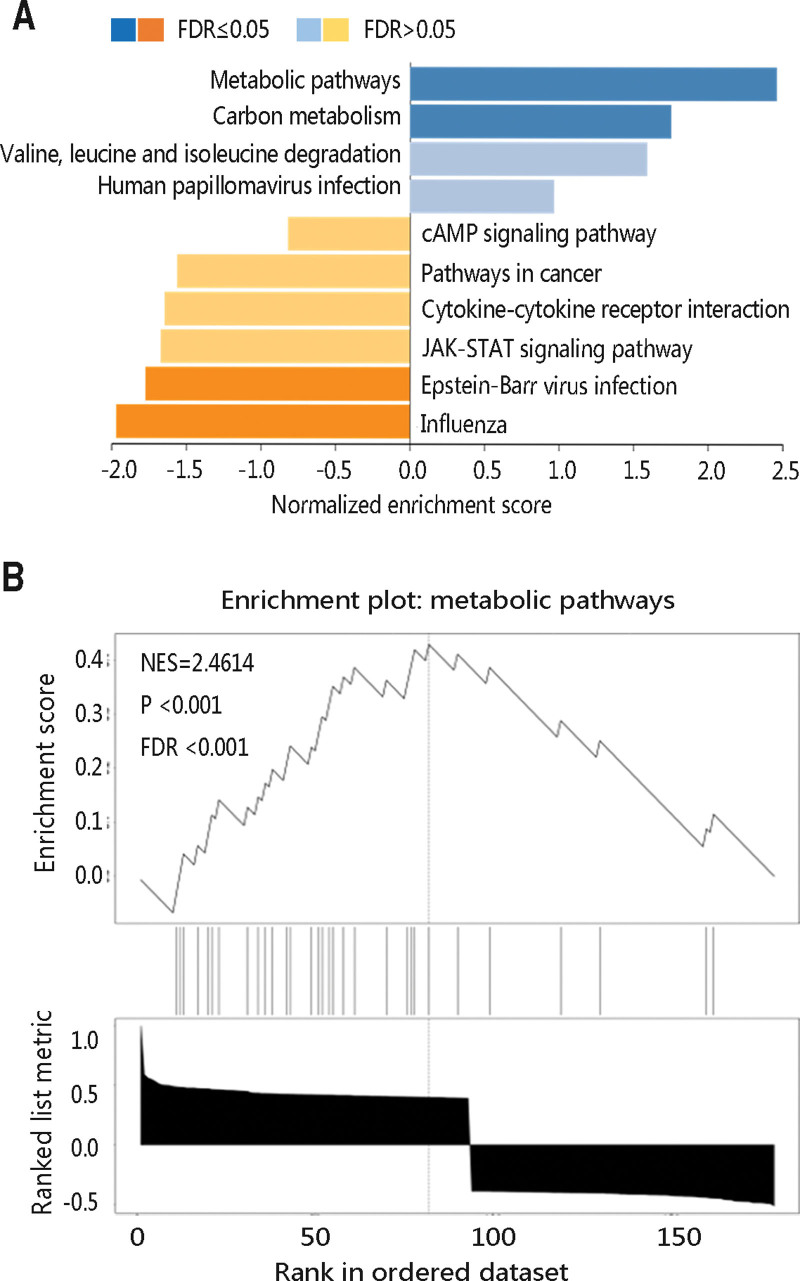
GSEA of THRSP and its co-expressed genes. (A) THRSP-related positive and negative pathways. (B) Metabolic pathways. FDR = false discovery rate, NES = normalized enrichment score, THRSP = thyroid hormone-responsive.

To investigate the underlying pathogenesis of THRSP in THCA, GSEA was performed. By setting the parameters, a total of 10 pathways were obtained (Fig. 7A), among which the metabolic pathway was the most enrichment pathway with a normalized enrichment score (NES) of 2.4614 (*P* < .001; Fig. 7B). As shown in Table 3, there were 3 positive significant pathways and 2 negative pathways (all *P* < .05). Of note, THRSP positively regulated the metabolic ways.

**Table 3:** THRSP-related positive and negative KEGG pathways in GSEA

KEGG name	ES	NES	Nominal *P* value	FDR *q* value
Metabolic pathways	0.42907	2.4614	<.001	0.000762
Carbon metabolism	0.52047	1.6839	<.05	0.047982
Cytokine–cytokine receptor interaction	−0.55491	−1.6377	<.05	0.061605
JAK-STAT signaling pathway	−0.6092	−1.672	<.05	0.067818
Valine, leucine, and isoleucine degradation	0.59195	1.5568	<.05	0.069307

### 3.6. PPI network construction on THRSP and KEGG analysis

**Figure 8.**
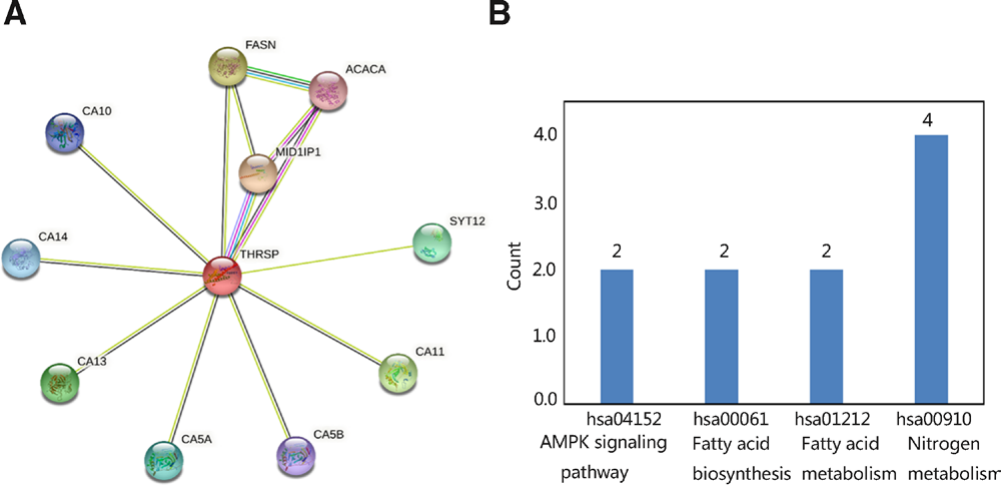
Protein–protein network construction and potential KEGG pathways of THRSP and its related proteins. (A) Proteins related to THRSP function. (B) Potential significant KEGG pathways. KEGG = Kyoto Encyclopedia of Genes and Genomes, THRSP = thyroid hormone-responsive.

Moreover, to determine the role of THRSP in THCA, the PPI network was constructed. Ten proteins were proved to be closely associated with THRSP protein, such as MID1IP1, FASN, and CA11 (Fig. 8A). The THRSP and its related proteins were mainly involved in metabolism-related pathways and the AMPK signaling pathway (Fig. 8B).

In view of the importance of THRSP for the prognosis of patients with THCA, this study mainly explored the pathways related to THRSP from 2 aspects. First, THRSP, together with its co-expressed genes were selected for enrichment analysis. Subsequently, the proteins associated with THRSP were generated for pathway analysis. Finally, we found that the pathways were concentrated in the metabolic pathways and THRSP had a positive correlation with the metabolic pathways. Based on the above findings, the authors inferred that THRSP positively regulated the metabolic pathways and inhibited the tumor progression, thus affecting the prognosis of patients with THCA.

## 4. Discussion

As a small nuclear protein, THRSP has been reported to play an important role in various cancers. Abnormal mRNA expression of THRSP was observed in breast cancer, kidney cancer, and liver cancer.^[25,26]^ We found that THRSP was lowly expressed in 12 kinds of tumor tissues, but highly expressed in THCA and KICH, which might result from the heterogeneity of cancer.^[27]^ In addition, the differential expression pattern is possibly due to the different epigenetic status of THRSP.^[28]^ Compared with adjacent normal tissues, THRSP mRNA expression was significantly higher in THCA tissues. The data from the Oncomine database confirmed the upregulation of THRSP in THCA. These findings triggered our interest to evaluate the prognostic value of THRSP in THCA. The GEPIA database analysis showed that high THRSP mRNA expression contributed to a favorable OS for patients with THCA. In hepatocellular carcinoma, downregulation of THRSP decreased epithelial markers and increased mesenchymal markers, hence facilitating epithelial to mesenchymal transformation (EMT) which is a critical process in tumor migration and invasion. Notably, THRSP suppressed the hepatocellular carcinoma cell proliferation, migration, and invasion by inhibiting the ERK/ZEB1 pathway induced by the EMT process.^[20,29]^ Moreover, THRSP overexpression in the MMTV-Neu transgenic model reduced mammary tumor metastasis compared to controls.^[30]^ The authors speculated that THRSP might inhibit THCA cell proliferation, migration, and invasion via inhibition of the EMT process; hence, the THCA patients with higher THRSP expression tended to have better clinical outcomes.

After characterizing the gene expression profile and the clinical significance of THRSP, we checked the mechanism underlying its dysregulation. Wells et al presented that low THRSP expression prolonged DFS of invasive breast cancer patients, but its expression did not differ between invasive cancers with or without lymph node metastases.^[15]^ Interestingly, we found that THRSP was significantly higher in THCA patients at the N0 stage than that at the N1 stage, indicating that THCA patients with high THRSP expression are less likely to develop lymph node metastases. This may also be the reason why the upregulation of THRSP led to the favorable prognosis of THCA patients. Available evidence has presented that gene expression was regulated by genetic alterations or epigenetic mechanisms, including amplification, deep deletion, mutation, and DNA methylation.^[31,32]^ DNA methylation has also been revealed to regulate cellular processes associated with tumorigenesis, providing a possibility to develop therapeutic targets by evaluating their DNA methylation.^[33]^ This study explored the genetic alteration and mutation of THRSP and the association of THRSP mRNA expression with DNA methylation. The results exhibited that mRNA high was the main form of genetic alterations which occurred in 3% of the patients. However, we failed to identify any mutation of THRSP in THCA. Notably, THRSP mRNA expression showed a strong negative relationship with its DNA methylation. Additionally, THCA tissues had a lower THRSP methylation level than the normal group. Therefore, the authors speculated that high THRSP mRNA expression might result from its low DNA methylation in THCA.

LNM had a close correlation with an unfavorable prognosis of thyroid cancer.^[7]^ Some clinical characteristics such as age, gender, and tumor size were identified as biomarkers for predicting LNM, but their functions are limited.^[34]^ Besides, the alterations of genes including BRAF and TERT have been proved to be associated with LNM, but with contradictory results.^[35–38]^ In this study, the increased expression level of THRSP was significantly related to low LNM risk through logistic regression analysis. Using the ROC curve for verification, THRSP showed good performance in predicting the LNM stage with an AUC of 0.70. Therefore, THRSP might serve as a valuable biomarker for predicting the OS and LNM stage of THCA patients.

To reveal the biological function of THRSP and its potential regulatory pathway, THRSP with the top 200 co-expressed genes were selected for the functional enrichment analysis. GO annotation results indicated that they were mainly enriched in cellular calcium ion homeostasis, cellular response to transforming growth factor-beta stimulus, extracellular exosome, an integral component of membrane, interleukin-1 receptor activity, and receptor activity. The major KEGG pathways included the biosynthesis of antibiotics, metabolic pathways, and carbon metabolism. GSEA further revealed that the most significant pathway was related to metabolism. Finally, the PPI network was constructed and the potential pathways in which THRSP and its related proteins participated were nitrogen metabolism, fatty acid metabolism, fatty acid biosynthesis, and AMPK signaling pathway. THRSP was involved in the fatty acid synthesis in normal and malignant mammary epithelial and adipose cells.^[13]^ The elevated levels of fatty acid synthesis were observed in multiple tumors since fatty acids as lipid precursors were used for membrane formation, protein modifications lipid posttranslational modifications, energy sustenance, and redox balance maintenance.^[39–41]^ In addition, certain fatty acids played a regulatory role in intracellular signaling pathways and were involved in the metastatic process.^[42,43]^ Thus, THRSP may be associated with the progression of THCA via positively regulating metabolic pathways, especially fatty acid metabolism.

This study had some limitations since it lacks in vitro and in vivo experiments to verify the mechanism of THCA occurrence and development. Nevertheless, we adopted efficient and reliable bioinformatics tools to identify the potential pathway in which THRSP might be involved, providing a direction for future basic experiments. Therefore, our study promotes further exploration of the THRSP gene in the future to deepen the understanding of the underlying mechanism of THRSP in THCA.

## 5. Conclusions

In conclusion, high THRSP expression was observed in THCA and its high mRNA expression predicted favorable OS in THCA patients. This is the first study to reveal the THRSP as a reliable predictor for the LNM stage. Moreover, THRSP inhibited the THCA progression through positive regulation of metabolic pathways, especially fatty acid metabolism.

## Author contributions

YZ and XC contributed to the conception and design. XS analyzed and interpreted the data. ZY contributed to the collection and assembly of data. All authors wrote and approved the final manuscript.
